# A technique to remove a well-fixed titanium-coated rm acetabular cup in revision hip arthroplasty

**DOI:** 10.1186/1749-799X-6-31

**Published:** 2011-06-20

**Authors:** Fernando MJ Judas, Rui F Dias, Francisco M Lucas

**Affiliations:** 1 University Clinic of Orthopaedics, FMUC – Faculty of Medicine, University of Coimbra. Praceta Prof. Mota Pinto, Bloco de Celas, 3000 Coimbra, Portugal

## Abstract

A major concern during revision hip arthroplasty is acetabular bone loss and bleeding during the extraction of well-fixed cementless acetabular cup, because no interface exists between the host bone and the cup. Forceful removal of such component using curved gouges and osteotomes often leads to extended bone loss and compromises reimplantation of a new socket.

In the following case report, we removed a well-fixed polyethylene titanium-coated RM acetabular cup with 20 years of follow-up, by significant wear of the polyethylene layer. The isoelastic femoral stem was also removed by mechanical failure.

We report a technique for removal of the cementless acetabular cup using powered acetabular reamers. The RM cup was sequentially reamed and when the polyethylene layer was thin enough, the remaining cup was removed easily by hand tools. The acetabular bone stock is preserved and the risks of bone fractures and bleeding are minimized. To our knowledge, these principles were applied only in cemented cups.

We have used this technique in 10 cases with excellent results and no complications were noted. This is a simple, reproducible, non-costly, non-timing consuming, safe and successful technique to remove well-fixed titanium-coated RM acetabular cups.

## Introduction

The purpose of revision hip arthroplasty is to reconstruct the hip to reproduce, as closely as possible, the form and function of the native joint. The indications for revision include significant polyethylene wear, fractures of the components, component malposition, hip instability, severe thigh pain, excessive damage to the femoral Morse taper, and severe infection [1-3].

In revision hip surgery, the removal of well-fixed cementless components can be extremely demanding, time consuming, and requiringes patience and caution to limit the amount of host bone destruction. Many surgeons opt to retain a well-fixed acetabular cup unless it is malpositioned or shows signs of impingement or severe wear. Fortunately, indications for implant removal are scarce, and most of the contemporary cementless components perform very well through improved bony ingrowth [4-6].

Because of the diversity of the components and the methods used to secure them, an equal diversity of approaches and tools are necessary for component extraction. Component removal in total hip arthroplasty revision is a crucial and essential step in the operation because it dictates the possibilities for component reimplantation and reconstruction, and therefore of the patient outcome. It is imperative that the surgeon is comfortable and familiar with the basic techniques of implant removal. On the other hand, the implant industry has responded accurately to the needs of the revision surgeon and developed instruments to overcome such issues with less effort [6].

We describe a technique to remove a well-fixed polyethylene titanium-coated RM cup using acetabular reamers, in a revision total hip arthroplasty. The reamers action reduces the thickness of the polyethylene to a thin lamina and this way the cementless cup can be removed easily, minimizing acetabular bone loss. The cup was removed by a significant polyethylene wear at 20 years of follow-up.

## Surgical Procedure

**Figure 1:**
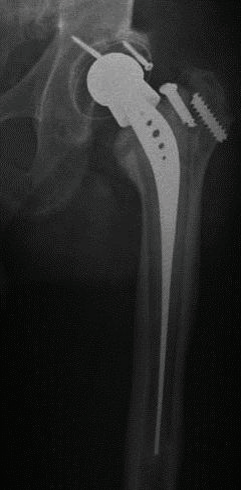
**Twenty years after primary hip arthroplasty**. The radiograph shows loosening of the isoelastic cementless femoral stem and well-fixed titanium-coated RM acetabular cup.

A 75-years-old man presented a bilateral hip arthroplasty. The clinic and the radiograph showed aseptic loosening of an cementless prosthesis in the left side, with 20 years of follow-up. The polyethylene titanium-coated RM acetabular cup was well-positioned and well-fixed, and no radiolucent lines are present in the bone/implant interface. The isoelastic femoral stem showed a mechanical instability, the two screws of stem being broken (Figure 1).

The hip arthroplasty revision was performed with the patient in the lateral decubitus position under general anesthesia, and the hip was exposed through a standard posterior approach.

The femoral implant was loose in the femoral cavity and was removed without difficulty. There were metallic debris in the soft tissues of femoral implant/bone interface.

The acetabular component was a monobloc hemispherical cup manufactured from Ultra High Molecular Weight Polyethylene (UHMWPE), with a pure titanium particle coated surface. With heat and pressure, the particles are blasted into the polyethylene surface. The coating promotes osseointegration. Stability of the cup is provided by two anchoring pegs on the weight bearing part on its outer surface. The inclination of pegs and holes diverges by 5 degrees providing a press-fit effect that increases the rigidity of the primary fixation and this is supplemented by screws inserted through the periphery of the cup [7,8].

The RM cup was removed, because a significant wear of the polyethylene surface was presented by direct observation and by placing an appropriately-sized head into the cup.

First we removed the three screws of the cup. The polyethylene layer of the cup was sequentially reamed using powered acetabular reamers.

**Figure 2:**
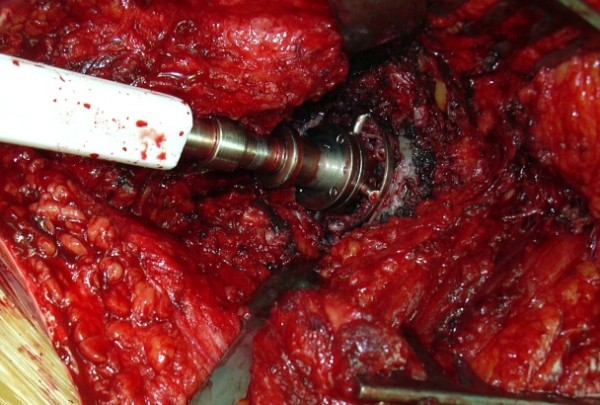
**Reaming of the center of the cup with powered acetabular reamers**.

**Figure 3:**
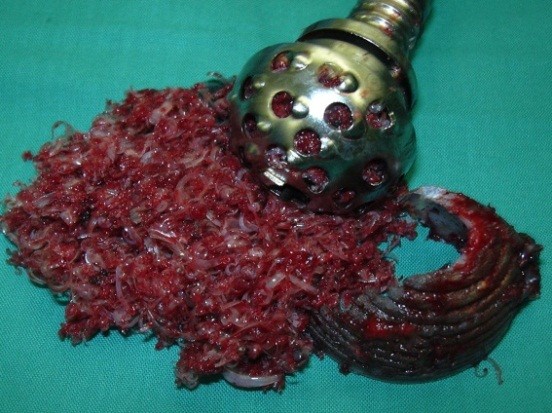
**When the polyethylene layer was thin enough, the remaining cup was removed easily by hand tools and bone stock is preserved**.

The first reamer was a small standard-powered *cheese grater *acetabular reamer (40 or 44 mm). It was placed in the middle of the inner part of the cup (Figure 2). Reamers of increasing sizes are used successively to wear the polyethylene layer (Figure 3). A minimal amount of polyethylene debris ("polyethylene chips") was removed of the articular soft tissues to avoid any biological reaction.

In this case, we used chisels to weak the superior part of the implant and to create a roughness surface for the reamer action. When the polyethylene was thin enough, it was removed easily by hand tools without the risks of bone fractures and bone stock loss. This technique is not time-consuming. The duration of the procedure was 12 minutes.

The cup surface was osseointegrated without interposition of soft tissues in the bone/implant interface, by a process of contact osteogenesis.

**Figure 4:**
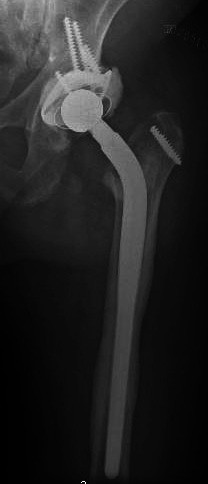
**Hip reconstruction with a metallic reinforcement ring and a conical cementless stem, without acetabular bone loss**. Cancellous bone allograft was used in the femoral side.

A metallic support ring and a cemented polyethylene cup were implanted. The femur was reconstructed with a revision conical stem and using morselized cancellous bone allograft cryopreserved [9-11]. The bone allograft was impacted to fill the space between the proximal part of the implant and the cortical native bone (Figure 4).

No complications were reported in the perioperative course and during the hospitalization period.

## Discussion

A major concern during revision hip arthroplasty is the acetabular bone loss during the extraction of well-fixed acetabular components. In fact, removing a well-fixed cementless cup is not always an easy task as it may lead to significant bone loss, particularly in the medial wall of the acetabulum, and subsequently result in a higher incidence of component loosening [12].

For safe component removal during revision hip procedures, it is essential to use the appropriate equipment and surgical techniques. During the last two decades, continuous efforts have resulted in the development of better tools to remove the cemented and cementless components. Hand tools such as osteotomes and chisels, ultrasonic devices, and ballistically driven chiseling systems controlled by an endoscope have been used with good results. There is no controlled study comparing the outcomes of the different tools and techniques, because often more than one device or approach is used simultaneously in the same revision procedure [1,2].

Removal of an ingrown fully porous-coated acetabular cup is a difficult procedure, because no interface exists between host bone and the cup. The procedure begins with complete acetabular rim exposure and extraction of the polyethylene. Because of the problems encountered in trying to replace an acetabular component associated to bone loss, surgeons have begun retaining the well-fixed component *in situ *by exchanging the polyethylene liner while attempting to encourage bone formation through grafting the defects with bone or bone substitutes [5]. This is typically done in elderly patients as a last resort.

If screws are present, it is imperative to have the appropriate screwdrivers available during the cup removal. A high-speed metal burr can be used to cut off the screw head if the screw is stripped or if difficulty is incurred during attempted extraction. In these situations we can use pliers, small trephines or screw removal instruments to remove the remaining part of the screw.

After adequate exposure the implant-bone interface is disrupted with the use of narrow curved osteotomes. Short curved osteotomes are used in a circumferential manner with gradual progression to longer osteotomes. Specific instruments have been developed and used to achieve the goal - safe component removal with minimal bone loss. The Explant™ system by Zimmer has been gaining popularity. This instrument allows safe penetration at the metallic cup-bone interface [6,12]. However, to remove a well-fixed titanium-coated RM acetabular cup the technique that we described is more efficient in preserving host bone.

In special situations, the metallic cup can be sectioned with a high-speed metal cutting burr. The sectioned component pieces are removed sequentially with minimal destruction of the underlying bone [1].

Despite the technology available and the numerous techniques described, the key element in component removal depends on a careful preoperative planning, technical expertise, and patience. Stepwise, logical progression can result in successful and complete removal of all components, whereas impatience can result in catastrophic bone loss and fracture [1,2,5,6].

In cases of well-fixed polyethylene titanium-coated RM acetabular component we can also use the general procedures recommended to removal of the cementless cups. However, there is always the risk of bone loss and bone fracture, because the cup is osseointegrated.

For that we would recommended to wear the polyethylene layer with powered acetabular reamers. When the polyethylene is thin enough, the remaining cup is removed easily by hand tools, despite the osseointegration of the cup surface. The acetabular bone stock is preserved and the risks of fractures and bleeding are minimized.

We have used this technique in 10 cases with excellent results and no complications were noted. To our knowledge and upon reviewing the literature, these principles were applied only in cemented cups [13].

## Conclusion

Removal of a well-fixed cementless titanium-coated RM acetabular cup can be associated with bone loss, bone fracture and bleeding, when we use curved gouges, osteotomes and chisels for disrupt the implant-bone interface. We have found the technique using acetabular reamers that allow cup thinning, to be simple, reproducible, non-costly, non-timing consuming and safe. The acetabular bone stock is preserved and the risks of complications are minimized. This technique can also be performed in the revision of a well-fixed all-polyethylene cemented cup.

## Competing interests

The authors declare that they have no competing interests.

## Authors' contributions

The lead author on this paper is FJ. He is an experienced hip surgeon, Chief of Service, Professor of the Faculty of Medicine, University of Coimbra, and he works in the University Clinic of Orthopaedics, Coimbra University Hospitals. The others authors are also experienced hip surgeons, Graduated Assistants in the University Clinic of Orthopaedics, Coimbra University Hospitals. They have participated in the surgery and contributed to the manuscript preparation. All the authors have read and approved the final manuscript.

## Ethical approval

The investigation process was developed in accordance with ethical research principles. The patient was informed that data concerning their case would be submitted for publication, and he consented.
